# Comparison of Low Resolution Electromagnetic Tomography Imaging Between Subjects With Mild and Severe Obstructive Sleep Apnea Syndrome: A Preliminary Study

**DOI:** 10.4306/pi.2008.5.1.45

**Published:** 2008-03-31

**Authors:** Hyun-Kwon Lee, Doo-Heum Park, Hyun-Sil Shin, Seok-Chan Hong

**Affiliations:** 1 Department of Psychiatry, Seoul National Mental Hospital, Seoul, Korea.; 2 Department of Psychiatry, Konkuk University School of Medicine, Seoul, Korea.; 3 Department of Otorhinolaryngology, Konkuk University School of Medicine, Seoul, Korea.

**Keywords:** Obstructive sleep apnea syndrome, Low-resolution electromagnetic tomography, Quantitative electroencephalography, Hypoxic brain damage

## Abstract

**Objective:**

The purpose of this study was to identify the regions of the brain associated with recurrent nocturnal chronic hypoxic episodes in patients with untreated obstructive sleep apnea syndrome (OSAS) using low-resolution electromagnetic tomography (LORETA) and quantitative electroencephalography (QEEG).

**Methods:**

Nocturnal polysomnograph (NPSG) and subsequent morning electroencephalograph (EEG) were measured in 20 subjects with OSAS. Mild (n=10 ages 39.5±12.1 years) and severe (n=10 ages 41.7±13.6 years) right-handed male OSAS subjects were selected by interview and questionnaires including the NPSG, Beck Depression Inventory, Beck Anxiety Inventory, Epworth Sleepiness Scale, and Pittsburgh Sleep Quality Index. The LORETA and QEEG were compared between the severe and mild OSAS groups by frequency bands (delta 1-3 Hz, theta 4-7 Hz, alpha 8-12 Hz, beta1 13-18 Hz, beta2 19-21 Hz, beta3 22-30 Hz, and total 1-30 Hz) made by spectral analysis during resting with the eyes closed.

**Results:**

The LORETA analysis showed decreased alpha activity at the right posterior cingulate gyrus (Brodmann area 23) in cases with severe OSAS compared to mild OSAS (p<0.05). For the QEEG, the absolute power of the alpha activity (8-12 Hz) was decreased in P3 (p=0.047), PZ (p=0.039) and O2 (p=0.04) in cases with severe OSAS compared to mild OSAS cases. The LORETA and QEEG analyses had similar results with regard to band, activation and location.

**Conclusion:**

The decreased activity of the alpha frequency in the right posterior cingulate gyrus, in patients with severe OSAS compared to those with mild OSAS, suggests that chronic repeated short-term hypoxia during sleep, in OSAS, could provoke cortical brain dysfunction associated with cognitive dysfunction such as memory and attention.

## Introduction

The obstructive sleep apnea syndrome (OSAS) causes repeated hypoxic episodes accompanied by arousal, due to upper airway obstruction, during sleep.^1^ The common symptoms of OSAS include frequent arousal during sleep and excessive daytime sleepiness,^2^ as well as an increased risk for the development of cardiovascular diseases such as hypertension, myocardial infarction and stroke,^2^-^4^ and psychological symptoms including depression and difficulties with interpersonal relationships, in addition to an increased risk of mortality.^5^-^7^ A Comprehensive meta-analysis from the neuropsych ological perspective revealed that untreated patients with OSAS had decreased executive function and vigilance, without affecting their overall cognitive, verbal and short-term verbal memory functions compared to normal control subjects.^8^

Research on the relationship between brain imaging findings and OSAS has contributed to understanding the physiopathology of OSAS.^9^ Imaging studies have shown that patients with OSAS have various brain lesions such as hippocampal atrophy^10^,^11^ and white matter lesions in the frontal lobes based on neurochemical studies using magnetic rosonance spectroscopy (MRS).^12^ In addition, functional brain imaging studies, to evaluate specific cognitive functions of patients with OSAS, have shown various lesions in the brain including decreased responses in the dorsolateral prefrontal cortex and increased responses in the frontal lobe, cingulate gyrus, thalamus, cerebellum, and the juncture of the parietal and temporal lobe, associated with certain tasks.^9^

The various physiological changes that occur with OSAS, as identified by neuropsychological examinations and brain imaging studies, suggest that the repetitive hypoxia due to upper airway obstruction,^13^ the sleep deprivation caused by recurrent arousals^14^ or an adaptive compensatory response^15^-^17^ cause the symptoms associated with OSAS. Although these brain imaging studies have contributed to the understanding of the neurophysiology of OSAS, our understanding of the pathophysiology of OSAS remains at a preliminary level. Further investigations using new neuroimaging methods are required in the future to improve our understanding of this disorder.^9^

Low resolution electromagnetic tomography (LORETA), used in this study, calculates the distribution of three dimensional electronic sources, showing the smoothest distribution, based on the hypothesis that a group of neurons with identical characteristics is fired and synchronized at the same time.^18^ The LORETA displays the distribution of current density, in solution spaces, divided by 2394 voxels of 7×7×7 mm on cortical gray matter and the hippocampus in the digitized probability atlas (Brain Imaging Center, Montreal Neurology Institute) based on the Talailach human brain atlas. Consequently, LORETA has an advantage for localizing the distribution of electrical sources of electroencephalograph (EEG) acquired bands from the cortical surface into three-dimensional space, despite its low spatial resolution.

In a study using the quantitative EEG (QEEG), patients with OSAS had a slowed EEG activity in the frontal lobe of the cerebral cortex compared to the normal control group, and it was reported to be associated with oxygen desaturation that developed during sleep at night.^19^ However, it is difficult to determine whether the EEG changes, in OSAS patients, were caused by recurrent hypoxic episodes, because of quantitative and qualitative sleep problems induced by frequent arousals in the OSAS patients compared to the normal controls. Therefore, it is necessary to analyze the EEG changes in OSAS patients with similar sleeping patterns, in quality and quantity, but a different severity of OSAS symptoms.

We hypothesized that chronic recurrent hypoxia, during sleep at night, in patients with OSAS is related to the changes in the EEG activity and the locations of the changes. Therefore, the goal of this study was to detect the specific bands and brain areas that are related to chronic brain hypoxia in association with OSAS. This was studied by determining the differences in the EEGs between groups with severe OSAS and those with mild OSAS using LORETA and QEEG techniques the day after a nocturnal polysomnograph (NPSG) was performed.

## Methods

### Subjects

The subjects were diagnosed with OSAS by their history, physical and neuropsychiatric examinations and NPSG. They were male patients with no other medical, neurological, psychiatric or sleep disorders, and were drug-free for two weeks or more before the examination. Twenty patients participated and they were all right-handed males. The subjects were divided into two groups: a group with mild OSAS (10 patients) with an apnea-hypopnea index (AHI) ranging from 5 to 15, and a group with severe OSAS (10 patients) with an AHI over 30. All subjects were evaluated for height, weight and blood pressure one hour before the NPSG, the Beck Depression Inventory (BDI),^20^ the Beck Anxiety Inventory (BAI),^21^ the modified Annett Handedness Scale,^22^ the Pittsburgh Sleep Quality Index (PSQI),^23^ and the Epworth Sleepiness Scale (ESS)^24^ were administered.

### Polysomnography

The Embla N7000 recording system (Medcare-Embla®, Iceland) was used for polysomnograph recordings and Somnologica version 3.3.1 (Medcare-Embla®, Iceland) was used for the analysis. The NPSG was carried out during one-overnight. The international standard for reading polysomnograph results was used for the sleep stages and events.^25^ EEG leads for sleep staging were applied at C3/A2, C4/A1, O1/A2, and O2/A1 using the international 10/20 system. Electrooculogram (EOG) leads were applied to the superior and inferior sides of the outer 1 cm surface at both outer canthi, as well as a chin electromyograph sensor on the submentalis muscle. A thermistor and nasal pressure cannula were used to determine airflow, a respiratory inductive plethysmograph to sense breathing movement on the chest and abdomen, and a pulse oximeter sensor to measure blood oxygen saturation on the left second finger. Apnea was defined as a cessation of airflow for at least 10 seconds and reduced airflow of more than 80%, and hypopnea as a cessation of airflow for at least 10 seconds and reduced airflow of 50 to 80%, accompanied by either a reduced blood oxygen saturation of more than 4% of basal oxygen saturation or arousal.^25^ AHI was defined as the number of episodes of apnea and hypopnea per hour.^26^

### Electroencephalograph measurement

EEG measurements were obtained the following morning after the NPSG, between 8-9 a.m. Thirty-two channels of the EEG were studied; (FP1, FP2, F3, F4, F7, F8, FT7, FT8, FC3, FC4, FZ, FCZ, T7, T8, C3, C4, CZ, TP7, TP8, CP3, CP4, CPZ, P3, P4, P7, P8, PZ, O1, O2, OZ) the reference leads were on both ears (M1, M2) and placed according to the international 10/20 system. The EEG measurements were obtained two times in the sitting position with the eyes-open and the eyes-closed for a total of 4 minutes each time, one minute alternatively in dim light. The examination room was electrically shielded, blocked from noise, and had the temperature and moisture controlled. The Neuroscan version 4.3.3 (Compumedics®, Australia) was used to measure the EEG results. In order to remove artifacts produced by the orbit and eyelid movements, vertical EOGs were applied at 1 cm from the outer portion of each eye and horizontal EOGs were applied at the upper and lower eyelids of the left eye. All impedance values were kept below 5 kΩ. The sampling rates were set to a 500 Hz/channel, sensitivity 0.15 uV, lower filter 0.05 Hz, high filter 60 Hz, time constant 0.3, and notch filter 60 Hz. The EEG epoch selected for LORETA was 6 seconds without artifacts with the eyes closed, which was averaged from three non-overlapping segments per epoch, containing 1,000 samples each. The EEG epoch for the QEEG was selected for 15 seconds without artifacts, with the eyes closed, which was averaged from five, three non-overlapping segments per epoch, containing 1,500 samples each. The EEG for the QEEG was analyzed by spectral analysis using the Neuroguide version 2.3.1 (Applied-neuroscience Inc, USA) software for EEG analysis.

### Electroencephalograph and statistical analysis

The EEG frequency bands were defined by delta (1-3 Hz), theta (4-7 Hz), alpha (8-12 Hz), beta1 (13-18 Hz), beta2 (19-21 Hz), beta3 (22-30 Hz) and the total (1-30 Hz). The source imaging was obtained by the LORETA-Key package.27 The errors caused by repeated measurement were corrected by a non-parametric independent analysis, the maximum t-statistic, for LORETA. The EEG results of specific areas of the brain, as defined by the voxel-by-voxel independent t-test, were compared between the two groups. In addition, the LORETA imaging of activated or decreased areas were evaluated. The LORETA imaging was converted to Talairach coordinates using the Talairach Daemon (TD) database, 1.0 version (Research Imaging Center, USA). For the QEEG analysis, Neuroguide version 2.3.1 (Appliedneuroscience Inc, USA) was used for the analysis; QEEG was calculated by a spectral analysis through fast Fourier transformation of the EEG. The absolute power of the QEEG was represented by the spectral power calculation of the real electrical power (µV^2^), and the electrode-by-electrode non-parametric independent analysis for these values was performed using the statistical package Neuroguide version 2.3.1 for comparisons of the two groups.

Data analyses were performed with SPSS 12.0 statistical software (SPSS, Inc., Chicago, IL, USA). A two-sided p<0.05 was regarded as statistically significant. The comparisons of the demographic and polysomnographic data between the two groups were analyzed by the Mann-Whitney test.

## Results

### Demographic and polysomnographic data

The participant's demographic characteristics such as age, education, body mass index, and systolic and diastolic blood pressure and the questionnaire survey of BAI, BDI, PSQI and ESS were compared between the two groups and no significant differences were identified (Table 1). For the NPSG, comparison between the two groups showed that the severe OSAS group had higher values for both the AHI and oxygen desaturation index (ODI)(p<0.001, p<0.001), and lower values for both the average SpO_2_ and the lowest SpO_2_ (p=0.011, p=0.009). The arousal index caused by apnea or hypopnea was significantly increased in the patients with severe OSAS (p=0.023), but there were no significant differences in spontaneous arousal, not due to apnea or hypopnea, between the two groups. In addition, the number of lower limb movements was greater in the severe group than the mild group (p<0.001). There were no significant differences in other sleep indices between the two groups (Table 2).

### Low resolution electromagnetic tomography source imaging and quantitative electroencephalography topography

**FIGURE 1:**
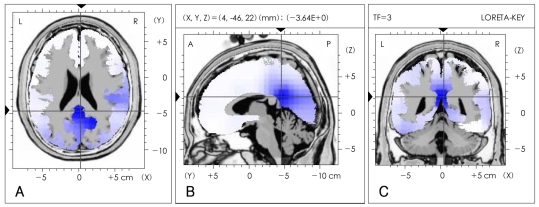
Comparison of low-resolution electromagnetic tomography (LORETA) source imaging of alpha (8-12 Hz) frequency bands between severe and mild obstructive sleep apnea patients. The LORETA image is displayed as the horizontal (A), sagittal (B) and coronal (C) sections showing the voxel with maximal t-statistic at the point of intersection (local maximal t-statistic -3.46, p<0.05). The blue area in the LORETA image represents decreased alpha (8-12 Hz) wave activity in severe OSAS patients compared to the mild OSAS patients. The point of intersection is right posterior cingulate gyrus (Brodmann area 23), which is the local maximal coordinates. The number and the volume of significant voxels associated with the point of intersection are 6 and 2.058 cm^3^, respectively. OSAS: obstructive sleep apnea syndrome.

**FIGURE 2:**
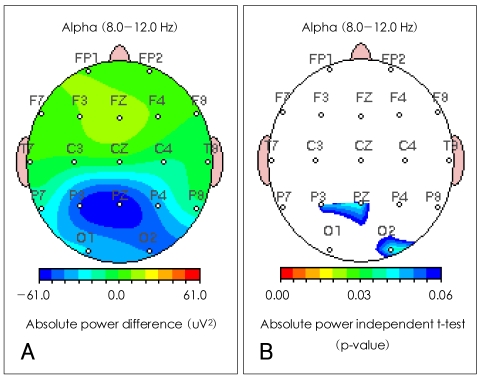
Comparison of quantitative EEG imaging of alpha absolute power between severe and mild obstructive sleep apnea patients. A: severe OSAS group compared to the mild OSAS group showed a significant decreases in P3 (-52.52 uV^2^), PZ (-60.34 uV^2^) and O2 (-41.51 uV^2^) in the absolute power differences of alpha waves. B: severe OSAS group compared to mild OSAS group showed significant p-values in P3 (p=0.047), PZ (p=0.039) and O2 (p=0.04) in the absolute power independent t-test of alpha waves. EEG: electroenceph-alograph, OSAS: obstructive sleep apnea syndrome.

Differences in the seven EEG frequency bands (delta, theta, alpha, beta1, beta2, beta3 and total) between the severe and the mild OSAS groups were analyzed by LORETA source and QEEG imaging techniques. The current density power was calculated from the LORETA imaging; the alpha waves were significantly decreased in the right posterior cingulate gyrus (Brodmann area 23)(p<0.05) in the subjects with severe OSAS compared to the mild OSAS subjects (Figure 1). However, the other bands were not significantly different in the comparisons between the two groups. The absolute power differences calculated from the QEEG imaging, in the severe OSAS subjects compared to the mild OSAS subjects, were significantly decreased at P3 (-52.52 uV^2^, p=0.047), PZ (-60.34 uV^2^, p=0.039) and O2 (-41.51 uV^2^, p=0.04) with no other significant differences in other variables between the two groups (Figure 2). When the decreased LORETA image of the right posterior cingulate gyrus was compared with the decreased QEEG image of P3, PZ and O2 from the alpha waves, the two images were noted to be similar in the bands, activations and locations.

## Discussion

This is the first study to determine the differences in EEG changes and activity locations according to the severity of OSAS using LORETA and QEEG. The hypothesis tested in this study was that the EEG would change based on the severity of hypoxic damage following chronic recurrent nocturnal hypoxia in patients with OSAS. Our findings showed that patients with severe OSAS, compared to those with mild OSAS, had significant alpha deactivation in the right posterior cingulate gyrus by LORETA imaging and at the P1, PZ and O2 sites by QEEG imaging.

In studies on EEG changes, induced by hypoxia, alpha deactivation has been consistently reported.^28^-^30^ In addition, reports have demonstrated that the limbic system is involved in controlling the respiratory neurons of the medulla oblongata, in hypoxic states, and respond to hypoxic stimulation.^31^-^33^ The results of our study showed that patients with OSAS had recurrent hypoxia. The differences between the two groups studied were noted in the posterior cingulate gyrus only. A prior study showed that verbal memory, constructional praxis and visual sustained attention could be reduced when the posterior cingulate gyrus is damaged by oxidative stress.^34^ In addition, previous investigations showed that information processing speed^35^ and attention^36^ are associated with alpha waves. Therefore, the results of this study are consistent with previous reports that demonstrated that patients with OSAS might develop cognitive dysfunction.^8^

QEEG is very sensitive to ischemic states.^37^-^40^ The patients with OSAS, compared to normal control subjects, have activated delta and theta waves in most cortical areas during wakefulness. The degree of activated slow waves has been shown to be positively related to the degree of oxygen desaturation on the NPSG.^19^,^41^ This suggests that brain damage caused by chronic and intermittent hypoxia, in patients with OSAS, can cause slow wave EEG changes. However, this study showed deactivation only in alpha waves with no changes observed in the slow waves. The explanation for our findings might be that our study group compared severe with mild OSAS, whereas most prior studies compared an OSAS group with normal controls. Frequent sleep fragmentation has been correlated with sleep deprivation. Reports have suggested that slow wave activation and alpha deactivation are present at wakefulness after sleep deprivation.^42^,^43^ Alpha wave reduction at wakefulness, after sleep deprivation, has been associated with a high motivation for sleep^42^; an increase in theta waves simultaneously with a decrease in alpha waves has been associated with a subjective feeling of somnolence.^42^,^44^-^46^ In a study on healthy people, during wakefulness after a day of sleep deprivation, the QEEG showed that the theta waves increased in areas T6, O2 and OZ, from relative power, and the delta waves in T6 areas, and the alpha waves decreased in areas Fp1, F3, Fp2, T4, T6, O2, and Oz from absolute power.^42^ Therefore, the findings of deactivation of alpha waves in severe OSAS compared to mild OSAS, in our study, may be associated with an increase in sleep fragmentation secondary to an increased level of micro-architecture following sleep apnea. However, some points contradict this inference. First, the two groups in our study showed no quantitative difference in sleeping stages, despite the fact that arousals due to apnea were more frequent in the group with severe OSAS than in the group with mild OSAS (Table 2). Second, our study showed no differences in the slow waves between the two groups. Third, the sites of alpha wave deactivation were present in the parietal lobe (P3, PZ) as well as in the occipital lobe (O2) unlike previous reports^42^ where it was in the fronto-temporo-occipital areas. Last, the ESS score for daytime drowsiness, though not significant, was lower in the severe OSAS than in the mild OSAS. However, it is possible that the alpha reduction observed in severe OSAS was caused by brain dysfunction, from hypoxic damage due to recurrent hypoxia during sleep, rather than daytime somnolence caused by sleep fragmentation or partial sleep deprivation.

This study had limitations including the following. First, the number of subjects was too small for definitive results. Thus, there should be further studies with a larger sample to confirm our findings. Second, the findings from this study could not explain completely the changes in brain electrophysiology in subjects with OSAS. Additional studies will be required to determine the differences in subjects with OSAS and those without this problem as well as comparing those with different severities of OSAS. Third, the analysis used in this study did not include the results of cognitive function tests associated with brain dysfunction due to OSAS. Fourth, even though LORETA has many advantages, it has limitations in pinpointing the areas affected by hypoxic damage. This is because LORETA imaging is based on 2,394 voxel areas related to the cerebral cortex and gray matter of the hippocampus.

In conclusion, despite the limitations, the results of this study demonstrate how to evaluate the pathophysiology of OSAS using LORETA. In addition, we presented a method that can be used to determine brain lesions caused by hypoxia in the patients with OSAS using the EEG, which is a cost-effective approach.

## Figures and Tables

**TABLE 1:**
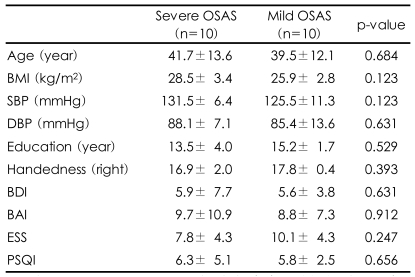
Demographic characteristics and questionnaire scores of patients with severe and mild OSAS

Results represent mean±standard deviation. OSAS: obstructive sleep apnea syndrome, BMI: body mass index, SBP: systolic blood pressure, DBP: diastolic blood pressure, BDI: Beck Depression Scale, BAI: Beck Anxiety Scale, ESS: Epworth Sleepiness Scale, PSQI: Pittsburgh Sleep Quality Index

**TABLE 2:**
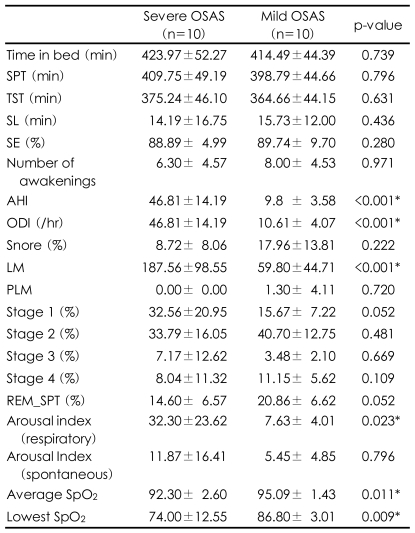
Polysomnographic data of patients with severe and mild OSAS

Results represent mean±SD. ODI was calculated by dividing the total number of oxygen desaturation (≥4% decrease in SaO_2_) by the total sleep time (hour). ^*^The values less than 0.05 of p-value. OSAS: obstructive sleep apnea syndrome, SPT: sleep period time, TST: total sleep time, SL: sleep latency, SE: sleep efficacy, AHI: Apnea-Hypopnea index, ODI: oxygen desaturation index, LM: leg movements, PLM: periodic leg movements
